# Their Economy and Our Health: Communicating Climate Change to the Divided American Public

**DOI:** 10.3390/ijerph17217718

**Published:** 2020-10-22

**Authors:** Haoran Chu, Janet Yang

**Affiliations:** 1 Department of Public Relations, College of Media and Communication, Texas Tech University, Lubbock, TX 79409, USA; 2 Department of Communication, University at Buffalo, the State University of New York, Buffalo, NY 14260, USA; zyang5@buffalo.edu

**Keywords:** climate change, psychological distance, issue framing, motivated reasoning

## Abstract

Climate change poses severe economic and public health threats to societies around the world. However, little is known about how selectively emphasizing its impacts on different issues and in different locations influence public engagement in climate change mitigation. Utilizing an experimental survey with adult participants, this study investigates the effect of issue framing and distance framing on risk perception and policy support related to climate change. The impacts of political ideology, environmental value, and belief in climate science on message effect are also examined. Based on the results of ANOVA (Analysis of Variance) and OLS (Ordinary Least Squares) regression, we found that compared with the economy frame, the public health frame led to greater polarization in risk perception and policy support between liberals and conservatives, and these relationships were mediated by environmental value and belief in climate science. Similarly, distance framing also increased ideological polarization in risk perception and policy support.

## 1. Introduction

Despite increasing global temperature and weather anomalies, public attitudes toward climate change are polarized in the U.S. [1,2,3]. In particular, conservatives are more likely to dismiss the threat of climate change and show less support for mitigation and adaptation policies [1,3]. As solutions to address climate change require bipartisan support, effective communication about climate change across ideological camps is critical [4]. However, people do not react uniformly to information even when it is backed by scientific evidence [5]. Research has long documented biases in people’s processing of scientific information [6]. Motivated by identity, value and prior belief, individuals often discredit information they disagree with and trust information they agree with [1]. This process, often called directional motivated reasoning, could lead to polarized opinion and a lack of unified action in addressing various social and political challenges, including climate change [1,7].

Message framing could be an effective strategy to overcome directional motivated reasoning in climate change communication, especially among individuals who hold skeptical opinion about the existence and cause of climate change [1]. Framing refers to the process of selectively emphasizing certain aspects of an issue, such as problem definition, causal interpretation, and treatment recommendation [8]. Research shows that framing can be an effective way to promote public awareness of climate change impact and increase climate change engagement, even among those who disagree with the science on climate change [1]. For instance, Schuldt, Konrath, and Schwarz found that simply referring to the phenomenon as “climate change,” rather than “global warming,” increased conservatives’ risk perception [9].

Two framing strategies that are especially relevant to this research are issue framing and distance framing. Issue framing refers to the selective emphasis on specific issues related to a larger phenomenon (e.g., different types of climate change impacts). Distance framing, on the other hand, highlights either far or close distance cues embedded in the overall narrative (e.g., climate change impacts in a faraway or nearby place). There is evidence that these framing strategies may be effective in reducing motivated reasoning based on ideological and partisan identity [10,11,12,13]. However, two important questions remain unaddressed. First, most studies only examine how partisans respond to climate change communication without explicating the motivational factors behind these responses [12]. As ideological camps are not internally homogenous groups [14], people upholding a particular political leaning may also be motivated by different factors when processing climate change information [10,13,15]. Therefore, it is meaningful to examine how political ideology, environmental value, and belief about climate change influence motivated reasoning. Second, although issue framing and distance framing could influence motivated reasoning, few studies have tested these two framing strategies simultaneously. As research has documented that public health frame could increase support for climate change mitigation at closer social distance [12], the next logical step is to examine whether similar results will emerge when a different type of issue is featured.

### 1.1. Motivated Reasoning

In an ideal world, people update their beliefs about the world by assimilating new information to form a more comprehensive understanding [1,6]. However, human information processing operates differently in reality. Instead of depositing new information into existing knowledge, people’s identity, value, and prior belief often influence the way in which they assimilate new information [7]. This goal-directed, biased processing of information is termed directional motivated reasoning [7]. Directional motivated reasoning often works through discrediting information that is incongruent with one’s identity, value or prior belief (i.e., disconfirmation bias) or seeking information that strengthen one’s existing belief (i.e., confirmation bias) [6]. For instance, Taber and Lodge found that both Democrats and Republicans disparage their rival party’s arguments on contentious issues such as affirmative action, while seeking evidence to support claims made by their own party [6]. As disconfirmation bias and confirmation bias could occur simultaneously, polarized opinion often ensues when partisans are exposed to the same information [6,15].

Over the past few decades, climate change has become a highly politicized issue in the U.S. [1,3]. Particularly, siding with party elites, conservative Republicans often dismiss scientific information on the cause and impact of climate change, while liberal Democrats are more likely to accept this information [10,16,17]. However, equating the conservative ideology with climate change denialism is an oversimplification because not all conservatives hold dismissive attitudes toward climate change [15]. Even among climate change deniers, the reasons behind motivated reasoning differ [10,18,19]. Therefore, it is important to explicate the underlying mechanisms that predispose conservatives to be more or less open to climate change communication [1]. As directional motivated reasoning is often driven by identity, value, and prior belief, this study addresses this query from these three angles [1,6]. Specifically, we study how these variables influence people’s response to climate change messages featuring different issues at varied distance. In the meantime, we acknowledge that these three factors are not the only determinants of motivated processing. We choose to study them because of their relevance to the current division in public opinion about climate change in the U.S. [1].

Various accounts have been offered to explain conservatives’ skepticism toward climate science. Some suggest that conservatives are prone to skepticism due to innate psychological characteristics such as intolerance of ambiguity and mental rigidity or closed-mindedness [19]. Others believe that conservatives are more likely to dismiss climate change because it is against their moral intuition [18,20]. Campbell and Kay, in comparison, argue that conservatism leads to skepticism because mitigation solutions, such as increased regulation is against the free-market value this group upholds [10]. Notably, the same study also points out that biased processing of scientific information is not unique to conservatives as liberals may discredit factual evidence that is incongruent with their views and beliefs as well [10].

In addition to the psychological characteristics and beliefs associated with conservatism, motivated reasoning may simply occur as a way to defend one’s political and ideological identity [21]. As ideology and partisanship often serve as identity markers [12], people may accept or dismiss claims simply based on who makes these claims [6]. For instance, people often follow political elites’ views on controversial social issues (e.g., climate change, gun control, and immigration) as a way to defend or strengthen their partisan identity [22]. This tendency may also serve as a way to reduce cognitive dissonance when encountering information that is incongruent with doctrines upheld by one’s own ideological camp [7,23]. Additionally, political ideology could serve as a heuristic cue for information processing when people take mental shortcuts to form opinion based on partisanship or ideology instead of facts or even personal experience [24].

Value is also an important motivator for confirmation bias or disconfirmation bias, which sometimes overwrites the influence of political ideology [1,10,21,25]. As illustrated earlier, Campbell and Kay found that conservatives, who tend to value free-market economy, are more likely to support climate change mitigation policies aimed at protecting the economy [10]. Similarly, Adger et al. found that conservatives are less likely to argue against climate change messages that underline moral principles that are valuable to them (e.g., purity, authority, and loyalty) [25]. Environmental value is a specific type of value that influences people’s decision making related to environmental protection and conservation [26,27]. It summarizes a person’s preference between environmental conservation and economic progress [27]. Environmental value is especially relevant to our discussion on climate change as economic progress is often achieved at the expense of the environment. For instance, the rapid post-war development around the world is largely powered by the fossil fuel industry [28], which also contributes to the exacerbation of climate change [29]. The economic consideration linked to environmental value is particularly relevant to our discussion, as conservatives usually uphold free-market value and care about economic development [10]. Therefore, liberals’ and conservatives’ different valuation of the environment vs. the economy is likely to influence their response to climate change communication.

Prior belief also determines the direction and level of motivated reasoning [1] over and beyond the influence of partisanship and ideology [15]. For example, conservatives who hold pro-environment attitudes are less dictated by the top-down party doctrine related to climate change [30]. Belief in climate science, including belief in the consensus among scientists on the anthropogenic nature of climate change, is a specific set of belief that could exert strong influence on conservatives’ response to climate change communication [31]. This belief has a critical influence on risk perception and policy support. On one hand, as climate change impacts are not always directly observable [32], people need to rely on science to determine whether natural disasters such as flood and drought are related to climate change. On the other hand, climate change’s most severe impacts are projected to occur in the future, and these projections are based on scientific simulation with different emission scenarios [33]. Thus, people’s support for mitigation policies may depend on their belief in climate science.

Notably, conservatives and liberals are quintessentially heterogeneous groups [10,13,15]. Therefore, delineating the underlying factors of motivated reasoning highlights the importance to look beyond political ideology as a single driver of their reactions to climate change communication. Supporting such argument, longitudinal observations of Americans’ attitudes toward climate change also show that public opinion is more complex than the two polarized views based on partisanship [34]. Therefore, pinpointing the influence of political ideology, environmental value, and belief about climate science is necessary to achieve a better understanding of how issue framing and distance framing influence people’s response to climate change communication.

### 1.2. Issue Framing

As a multifaceted problem, climate change affects various aspects of our society. Two issues of particular interest are public health and the economy, as they are highly relevant to people’s wellbeing regardless of political or cultural identities [31,35]. As illustrated earlier, some research suggests that emphasizing climate change’s impact on public health could alleviate ideological polarization [4]. However, other studies have shown that conservatives sometimes discredit communication messages that link climate change to public health concerns [12,36]. In contrast, highlighting the economic impact of climate change seems to more consistently reduce counterarguing among conservatives because conservatives’ core value associated with a free-market economy may contribute to this reduced polarization [10,21].

These two types of issues also differ in other aspects. Notably, public health issues often involve diseases and epidemics that can instigate strong negative emotions, while economic issues may be affect-neutral or even generate positive emotions. For instance, images related to health often generate negative feelings while those of artifacts related to the economy tend to instigate more neutral or even positive feelings [37]. Political psychology research shows that political ideology may be linked to people’s different biological tendency to respond differently to negatively-valenced stimuli [38,39]. Particularly, as higher sensitivity to negative stimuli may be more closely associated with conservative than liberal beliefs, and people holding conservative ideology may also show stronger tendency to engage in avoidance regulatory strategies in response to dangers and threats [40,41].

Correspondingly, negatively-valenced imageries may trigger more biased information processing among conservatives and generate more counterarguing. Empirical research corroborates this claim. For instance, Feinberg and Willer [42] found that presenting the dire consequences of climate change (i.e., negative stimuli) significantly reduced people’s confidence in its existence, especially among those who uphold a strong belief in a just world, an important characteristic of the conservative identity [14]. As climate change mitigation policies may be seen as unfair for certain social groups (e.g., those who have to pay a higher carbon tax), conservatives who strongly endorse the fairness and justice moral foundations may find negatively-valenced imageries highlighting climate change impacts less palatable [14]. Similarly, McCright and colleagues [31] found that compared to economy and national security frames, presenting climate change as a public health issue failed to compete against climate change denial messages in promoting positive attitudes toward climate change mitigation. Based on these theoretical arguments and empirical evidence, we argue that ideological polarization would decrease when climate change impacts are featured as a threat to national economy as compared to a threat to public health.

As mentioned earlier, value and prior belief are also likely to determine the direction and magnitude of motivated reasoning, sometimes exceeding the influence of ideology [1]. For example, research shows that accounting for ideology, only those who hold extreme attitudes on specific issues such as gun control generate skeptical arguments against political messaging [6]. Similarly, ideological polarization related to climate change may also be influenced by environmental value and belief in climate science, which are important determinants of people’s attitudes toward climate change mitigation [27,31,43]. Particularly, since liberals and conservatives may hold different environmental value and belief in climate science, it is meaningful to examine the influence of these two variables on ideological polarization.

### 1.3. Distance Framing

Presenting climate change as a distant or close threat could also instigate different responses from people with different political ideology [11,44,45]. Specifically, portraying climate change impacts at increased psychological distance often leads to greater ideological polarization in risk perception and behavioral intention [11,46]. For instance, the gap between conservatives’ and liberals’ support for climate change mitigation policies increased when participants read about climate change impacts on a public health issue in a foreign country [12]. Chu and Yang [11] also demonstrate that ideological polarization is more pronounced when climate change impacts are portrayed as influencing distant others. Based on these findings, it is arguable that polarization in risk perception and policy support will decrease when climate change impacts are portrayed as influencing the U.S. as compared to a foreign country.

It is worth noting that distance framing is also relevant to climate change communication targeting the U.S. public, especially among Americans who reside in areas where climate change impacts are less noticeable. Specifically, as climate change has a greater impact on developing countries, people in developed countries may be less likely to perceive it as a personally relevant issue [32]. Exacerbating the situation, media coverage on climate change often features imageries from distant locations such as polar bears drifting on ice [32]. Thus, explicating the influence of distance framing on ideological polarization also has important practical value.

Different explanations have been offered to account for distance framing’s impact on ideological polarization. For example, construal level theory suggests that people tend to form more abstract mental representations of an object or an event that is far away from them [47]. Thus, their subsequent attitudinal and behavioral responses are also influenced more by the abstract, “high-level” features associated with the object or event [48,49]. Identity, value, and prior belief are high-level mental construal that tend to have consistent impacts on people’s perception and judgment across different contexts [2,6,11,49]. Thus, they are likely to have a stronger impact on people’s reactions to climate change impacts portrayed at greater psychological distance (e.g., flooding in a foreign country). Supporting this argument, Ledgerwood et al. found that ideology’s impacts on people’s opinions about immigration policies are more pronounced at far social distance than at close social distance [49].

Other researchers argue that increased distance results in weaker identification with climate change victims, which leads to decreased support for mitigation policies among conservatives [12]. As distancing climate change impacts from one’s familiar surrounding may lead people to view these impacts as someone else’s problem, people who hold more dismissive attitudes toward climate science or less pro-environment value may be less likely to support climate change mitigation at increased distance. Therefore, it is possible that risk perception and policy support may further diverge between conservatives and liberals upholding different environmental values and beliefs in climate science.

### 1.4. Current Research

**Figure 1:**
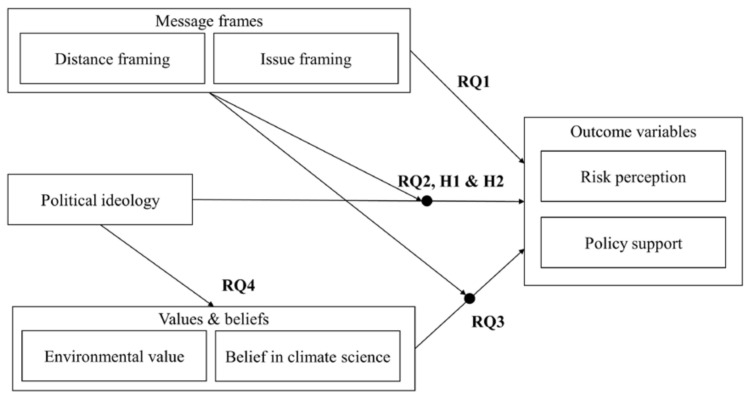
Theoretical framework of the current study with hypotheses and research questions.

Considering that both issue framing and distance framing are likely to increase ideological polarization, it is possible that issue framing and distance framing may interact to influence ideological polarization, in a way that the public health issue positioned at far distance may lead to the largest polarization. Thus, this study investigates how political ideology, environmental value, and belief in climate science motivate different responses to climate change communication. Specifically, we first examine if issue framing and distance framing influence participants’ risk perception and policy support (RQ1). Further, we test if issue framing and distance framing have different impacts on conservatives and liberals (RQ2). We hypothesize that the gap between conservatives’ and liberals’ risk perception and policy support (i.e., ideological polarization) would be larger when participants are exposed to far-distance (H1a) or public health message (H1b). Further, we test if the public health frame positioned at far distance leads to the largest polarization (H2). Lastly, we examine if environmental value and belief in climate science also lead to different risk perception and policy support (RQ3), and whether these differences contribute to political polarization (RQ4). Figure 1 summarizes the theoretical framework and research questions.

Corresponding to the research questions, we conducted a 2 × 2 full-factorial experiment. Participants in each condition viewed an animated video highlighting climate change impact on a public health issue (i.e., babesiosis) or an economy issue (i.e., coffee) in the U.S. or in Indonesia, a country that is socially and geographically distant to our U.S.-based participants. Political ideology (i.e., liberal, middle-of-the-road, or conservative), environmental value, and belief in climate science were pre-test measures; risk perception and policy support were post-test measures.

## 2. Materials and Methods

The study has been approved by the Institutional Review Board at University at Buffalo, the State University of New York (IRB ID: STUDY00001439). Participants provided informed consent by reading and agreeing to a consent form at the beginning of the questionnaire. Psychological and cognitive factors such as environmental values and belief in climate science were measured before exposure to the stimuli, and outcome variables were measured post-test. Political ideology was measured with demographics toward the end of the survey. The data were analyzed anonymously.

### 2.1. Sample

A U.S.-based adult sample (N = 950) was recruited on Amazon MTurk from late July to early August in 2017. We specified U.S. location and past approval rate of greater than 95% as qualifications for MTurk workers. The survey was hosted on Qualtrics.com [50]. As an attention check, participants were asked to choose what was mentioned in the video they just watched from four options including coffee, babesiosis, maple syrup, and malaria. Those (n = 96) who failed to identify the correct exemplars were excluded from subsequent analyses, resulting in a sample of 854. Pearson’s Chi-square test indicates that there were no significant between-condition differences in participants who failed the attention check and those who passed (*χ*^2^_(3)_ = 1.66, *p* = 0.65). The passing rate (89.9%) also resembles past research that shows MTurk workers are often more attentive than respondents in other convenience samples such as college students [51]. Though Amazon MTurk workers in general are more liberal-leaning [52], research indicates that the ideological disposition of participants recruited from this platform faithfully mirror those of the general population such that conservative Amazon MTurk workers do not differ significantly from their off-the-platform counterparts [52].

The majority of our participants were female (54.2%). The average age was 36.69 (SD = 12.23), with a majority self-identified as Democrat (46.5%), followed by Independent (32.9%) and Republican (20.5%). Median annual household income fell into the bracket of US$50,000–$74,999, and the majority have obtained a bachelor’s degree or higher (53.6%). Results of a series of univariate analyses of variance (ANOVA) and Chi-square tests indicate that there were no significant between-condition differences in demographics. Therefore, random assignment was successful.

### 2.2. Stimuli

Four animated videos were created on PowToon.com, an online platform for animation production, to illustrate the respective impact of climate change in the U.S. or in Indonesia (links to video stimuli can be found in the Appendix A). The public health frame stresses climate change’s impact on Babesiosis, a potentially life-threatening tick-borne disease, including increased hospitalization due to Babesiosis infection. The economy frame features climate change’s impact on the coffee industry, including reduced production and decreased revenue. Both exemplars are realistic to the U.S. and Indonesian settings. Babesiosis infection has been observed in both countries. While coffee is a major agricultural commodity in Indonesia, it is a major consumer good in the U.S. All videos (each is about 57 s in length) include a general introduction to climate change, and they are narrated by a native English speaker.

### 2.3. Measures

#### 2.3.1. Political Ideology

Political ideology was measured with one-item (“when it comes to politics, you generally consider yourself to be”) along with demographics toward the end of the survey. Participants’ response to this question was assessed with a 7-point scale ranging from very conservative (1) to very liberal (7). As expected, the MTurk sample was more liberal leaning (M = 4.62, SD = 1.83), with 247 participants (28.9%) considered themselves as conservative (including those self-identified as extremely conservative, conservative, independent but leaning toward conservative), 475 self-identified as liberal (55.6%; including extremely liberal, liberal, and independent but leaning liberal) and 131 holding a middle-of-the-road ideology (15.3%).

#### 2.3.2. Environmental Value

Environmental value and belief in climate science were measured in the same block before participants watched the video stimuli. All scale items were counterbalanced to avoid ordering effect. Two items gauged participants’ valuation of environmental conservation versus economic progress [26]— “We worry too much about the future of the environment, and not enough about prices and jobs today” and “People worry too much about human progress harming the environment”. Participants’ responses were assessed on a seven-point scale (1 “Strongly disagree” to 7 “Strongly agree”). After recoding, higher value of the composite score indicated preference for environmental conservation over economic progress. The scale was reliable (α = 0.86) and our participants on average showed higher valuation of economic development over environmental conservation (M = 3.84, SD = 1.09).

#### 2.3.3. Belief in Climate Science

Belief in climate science was measured with a scale adopted from McCright et al. [31]. The scale has five items, including “The scientific evidence that climate is changing is very solid’, “The scientific evidence that climate is changing because of human activities is very solid”, “Claims that the climate is changing are based more on politics than on science”, “Many scientists do not believe the climate is changing”, and “Claims that the climate is changing are based more on politics than on science”. The last two items were reverse-coded. Participants provided response on a 7-point scale (1 “Strongly disagree” to 7 “Strongly agree”). A composite score was created after recoding some items so that higher score indicated stronger belief in climate science (M = 5.46, SD = 1.53, α = 0.90). In general, our participants reported a firm belief in climate science.

#### 2.3.4. Risk Perception

Risk perception and policy support were measured after participants’ exposure to the stimuli. These items also appeared in random order. Three items measured risk perception on a 5-point scale (1 “Not at all likely” to 5 “Extremely likely”). Participants were asked to indicate perceived risks of climate change on themselves and their community, future generation, and people all over the world (“how much do you think climate change will harm …”) [11,44]. Upon reliability check (α = 0.93), responses to the questions were averaged to create a composite scale of risk perception (M = 4.03, SD = 1.06). Overall, participants reported high risk perception about climate change.

#### 2.3.5. Policy Support

Participants’ support for five climate change mitigation policies were measured on a 7-point scale (1 “Strongly oppose” to 7 “Strongly support”; “the following policies have been proposed to reduce the impact of climate change in cities around the world. Using the scale provided, please indicate how much you support or oppose these policies”). These policies include “regulate carbon dioxide (the primary greenhouse gas) as a pollutant,” “require automakers to increase the fuel efficiency of cars, trucks, and SUVS to 54.5 mpg,” “require electric utilities to produce at least 20% of their electricity from wind, solar, or other renewable energy sources,” “fund more research into renewable energy sources, such as solar and wind power,” and “provide tax rebates for people who purchase energy-efficient vehicles or solar panels.” [53]. The scale was reliable (α = 0.91), and most participants were fairly supportive of mitigation policies (M = 5.74, SD = 1.35). Of note, although these policies may require increased governmental regulation, which contradicts the free-market value upheld by many conservative Republicans [10], they were utilized for two reasons. First, they are widely used in previous research, which allows us to compare our findings against existing literature. Second, these items may allow us to detect the differences between liberals’ and conservatives’ policy support more clearly.

## 3. Results

**Figure 2:**
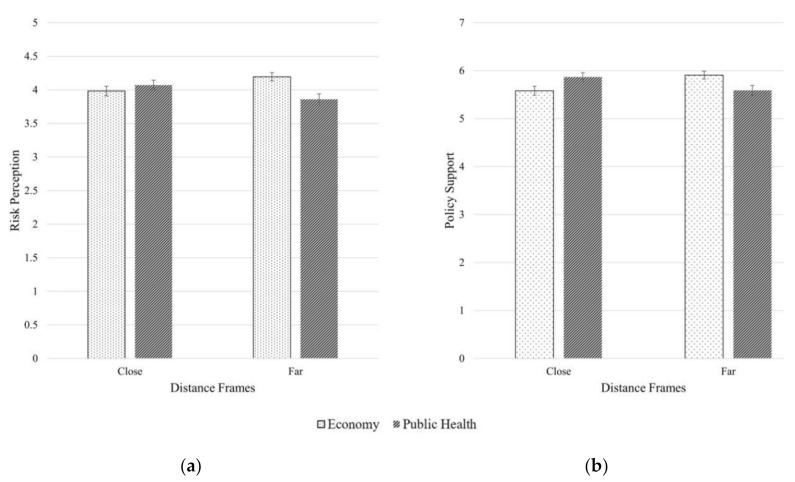
Issue framing and distance framing interact to influence risk perception and policy support. (**a**) Perceived risk of climate change reported by participants assigned to four experimental conditions; (**b**) Support for climate change mitigation policies reported by participants assigned to four experimental conditions.

In response to RQ1, two-way ANOVAs were utilized to examine the direct effects of issue framing (i.e., economy vs. public health) and distance framing (i.e., U.S. vs. Indonesia) on risk perception and policy support. Issue framing did not have significant main effect on risk perception (F (1, 850) = 0.03, *p* = 0.87) or policy support (F (1, 850) = 2.96, *p* = 0.09). Distance framing also had no significant main effect on risk perception (F (1, 850 = 0.06, *p* = 0.81) or policy support (F (1, 850) = 0.00, *p* = 0.99). However, there were significant interaction effects between the experimental factors on risk perception (F (1, 850) = 10.76, *p* < 0.01) and policy support (F (1, 850) = 8.75, *p* < 0.01). Simple slope analyses revealed that the economy frame outperformed the public health frame in increasing risk perception at farther distance (F (1, 421) = 11.11, *p* < 0.01), but not at closer distance (F (1, 429) = 0.76, *p* = 39). Compared to the public health frame, the economy frame also led to higher policy support at farther distance, F (1, 421) = 5.83, *p* < 0.05, and lower policy support at closer distance, F (1, 429) = 4.94, *p* < 0.05. Figure 2 illustrates these interaction effects.

Corresponding to RQ2 and H1–H2, which enquire about message effect on ideological polarization (i.e., greatest difference in risk perception and policy support between liberals and conservatives when public health frame is positioned at far distance), OLS regression analyses were conducted with risk perception and policy support as outcome variables and message frames, political ideology, and their interactions as predicting variables. Issue framing (0 = economy; 1 = public health) and distance framing (0 = close; 1 = far) were dummy coded to facilitate the interpretation of results. Results of these analyses are reported in Table 1.

Observably, political ideology (higher value indicating more liberal ideology) is a significant predictor of both risk perception and policy support, indicating that liberals are more likely to perceive climate change as a substantial risk and support mitigation policies. Similar to the ANOVA results, issue framing and distance framing interacted to influence risk perception and policy support. The public health frame positioned at far distance led to the lowest level of risk perception and policy support.

**Figure 3:**
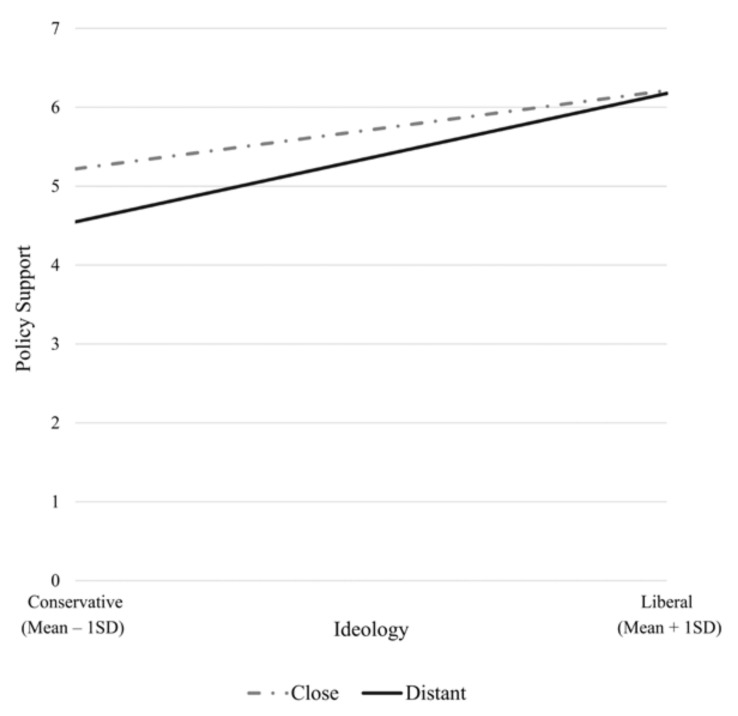
Distance framing and political ideology interacted to influence polarization.

The three-way interaction among issue framing, distance framing and political ideology was also significant in predicting policy support. To further probe this interaction, we ran four regression models. The first two models included participants from either close distance or far distance conditions. Issue framing, political ideology, and their interaction were included as predictors and policy support was the outcome variable. The other two models included participants in the economy or public health conditions, with distance framing, political ideology, and their interaction as predictors, and policy support was the outcome variable. The only significant interaction observed was between ideology and distance framing for the public health messages (B = 0.16, *p* < 0.05). This result suggests that reading about climate change’s impact on public health in a foreign country led to a bigger gap in policy support between liberals and conservatives (Figure 3). Spotlight analysis with Johnson–Neyman method indicates that the public health frame anchored at far distance was particularly unappealing to conservatives (lower 45.31% of ideology score).

Corresponding to RQ3 (interactive effects between message framing and environmental value or belief in climate science), two sets of OLS regression analyses were performed. The regression models followed a similar setup as shown in Table 1. For the first set of models, dummy-coded issue framing (0 = economy; 1 = public health) and distance framing (0 = close; 1 = far), environmental value, and the interaction terms were included as predictors (Table 2). In the second set of models, framing, belief in climate science and interaction terms were included as predictors (Table 3). Similar to the findings detailed above, significant interaction between issue framing and distance framing indicated that the public health frame positioned at far distance consistently led to lower risk perception and lower policy support. As expected, environmental value and belief in climate science were both significant predictors of risk perception and policy support. That is, participants who valued environmental conservation over economic progress and participants who strongly believed in climate science reported higher risk perception and policy support.

**Figure 4:**
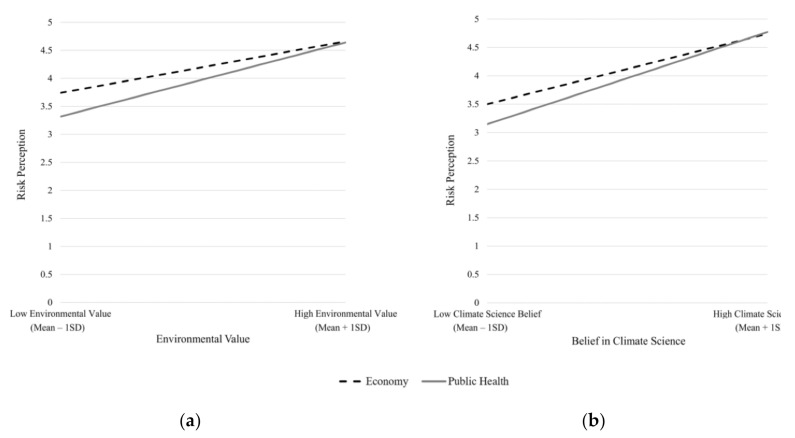
(**a**) Issue framing interacts with environmental value to influence risk perception; (**b**) Issue framing interacts with belief in climate science to influence risk perception.

Three-way interaction between the two frames and environmental value or belief in climate science were also significant predictors of risk perception. We ran eight regression models to probe these significant interactions. The only significant two-way interactions were between issue framing and environmental value (*B* = 0.20, *p* < 0.01) and between issue framing and belief in climate science (*B* = 0.13, *p* < 0.05) among participants who read the far-distance messages. Closer inspection of these interactions with Johnson–Neyman method indicated that at far distance, those who valued economic progress (lower 58.16% of the environmental value scale) and those who were skeptical about climate science (lower 46.57% of the science belief scale) were less likely to perceive risk from climate change when exposed to the public health frame. Figure 4 illustrates these interactions.

In response to RQ4, which asks if differences in environmental value and belief in climate science contribute to liberals’ and conservatives’ different responses, two moderated-mediation analyses were conducted with PROCESS [54]. Political ideology was introduced into the model as the independent variable, environmental value and climate science belief were mediators, issue framing and distance framing were moderators, and risk perception and policy support were dependent variables. Table 4 and Table 5 show the bootstrapped direct and indirect effect of ideology on risk perception and policy support via the mediation of environmental value or belief in climate science. Observably, the impacts of political ideology on risk perception and policy support were fully mediated by environmental value in all but the economy/far distance condition. Liberals and conservatives showed different levels of risk perception and policy support after viewing the video on climate change’s impact on the coffee industry in Indonesia due to their different political ideology, not because they valued environment conservation more than economic development. The impacts of political ideology on risk perception and policy support were fully mediated by belief in climate science in all conditions.

## 4. Discussion

### 4.1. Issue and Distance Framing

Results from this study replicate findings from previous research [4,12] by demonstrating that highlighting distant public health impacts of climate change may backfire among people who hold more dismissive attitudes toward climate change. Corresponding to our first research question, we found that the portrayal of climate change impact on the U.S. (vs. Indonesia) increases participants’ risk perception and policy support, and this effect is stronger when framed as a public health issue (vs. an economy issue). In contrast, the economy frame outperforms the public health frame in increasing risk perception and policy support at increased distance. These findings suggest that issue framing and distance framing need to be presented strategically to promote climate change engagement. In particular, stories on climate change impact on the economy may be more effective when presented at far distance, while impact on public health may be more effective when positioned at close distance. The interplay between issue framing and distance framing pushes the boundary in existing literature, which either focuses on issue framing (e.g., national security vs. public health [4]) or on distance framing [12]. Because the utility of one framing strategy may be amplified or attenuated by other factors, as illustrated in this study, researchers and practitioners should consider incorporating more than one strategy when communicating to diverse audiences about climate change.

### 4.2. Message Framing and Motivated Reasoning

The second and third research questions enquire about polarization based on political ideology, environmental value, and belief in climate science. Observably, liberals and conservatives diverge more in their support for climate change mitigation policies when the public health frame is positioned at far distance. In contrast, compared to emphasizing climate change impact on the economy, linking climate change impact to a public health issue leads to greater polarization in risk perception among individuals who hold different environmental value and belief in climate science, especially at far distance. Together, these results suggest that the emphasis on a distant location may have led participants to construe climate change impact as a more abstract, high-level phenomenon [47], which means they may have relied more on political ideology, environmental value, and existing belief in climate science to form risk judgment and policy support about climate change. This finding supports research related to the construal level theory of psychological distance [11,48,49].

Interesting results also emerge from the moderated-mediation analyses corresponding to our last research question on the relationship among political ideology, environmental value, and belief in climate science. The economy-far condition stands out because liberals and conservatives report different risk perception and policy support in this condition primarily due to their different belief in climate science, not environmental value. As the economy frame speaks directly to conservatives’ valuation of economic progress over environmental conservation [10,14], it is not surprising that environmental value does not mediate the relationship between political ideology and the outcome variables in this particular condition. Compared to the other two conditions, political ideology also has a smaller impact on risk perception and policy support in the close-economy condition. Together, these findings suggest that when people’s fundamental value (i.e., a conservative political ideology) and message framing (i.e., climate change impact on the economy) align, they are more likely to rely on their political ideology, rather than environmental value, to form opinion about climate change.

Congruent with existing research, liberals and conservatives report different risk perception and policy support [2,6,11]. However, these differences become more polarized when the public health frame is positioned at far distance. Additionally, political ideology seems to operate along with environmental value and belief in climate science in influencing risk perception and policy support. These findings shed some light on climate change communication practice. First and foremost, focusing on climate change impact on public health in a foreign land is not an effective strategy to engage the political conservative. In contrast, due to their penchant for economic prosperity [12,14,19], emphasizing climate change impact on the U.S. economy may be more persuasive if the goal of communication is to increase risk perception and policy support among a conservative audience. Our findings also highlight the importance of fostering public understanding and trust in the science on climate change because belief in climate science has the potential to reduce political polarization.

### 4.3. Limitations and Recommendations for Future Research

Like all research, this study has limitations. First and foremost, the use of MTurk sample reduces the generalizability of our findings. Consistent with previous research, MTurk workers are more liberal leaning, which means we had limited access to conservative participants. Considering that political ideology is central to climate change communication, future research needs to oversample conservatives when using the MTurk platform. Further, as only participants living in the United States were sampled and we did not assess participants’ immigration status or country of origin, we recommend future research to recruit a more diverse sample and assess the possible influence of culture and nationality on people’s response to climate change messaging. Additionally, with concerns over the quality of MTurk data, future research needs to replicate the relationships observed in this study using a more representative sample.

Other limitations are related to measurement and the analytical approach. Specifically, political ideology was measured with a single item. Notably, as ideology was conceptualized as an identity endorsed by the respondents, this item may eliminate further consideration related to partisanship. Although this is a common approach to measure political ideology [14], future studies may consider employing more complex measures such as assessing participants’ attitudes toward specific social and economic issues [38]. In addition, tighter governmental regulation was inherent in several of our policy support measures. Future research should explore including climate change mitigation policies that are more appealing to a conservative audience, such as cap-and-trade [10]. Furthermore, environmental value and belief in climate science were entered into the moderated mediation models as mediators, but their relationships with ideology were correlational. Though similar correlational relationships were analyzed with mediation models in existing research [10], it limits this research’s capability to establish causality. In a similar vein, assessing these variables prior to the experimental stimuli may have primed participants on their climate change beliefs. Although random assignment may have ensured the integrity of our main results, we recommend future research to test these relationships with more nuanced experimental design.

Another limitation pertains to the observational equivalence of accuracy and directional motivated reasoning suggested by Druckman and McGrath [1] in their recent review on motivated reasoning research in the climate change context. The authors suggest that conservatives may not always slant climate change information to defend identity, value and prior belief, they may simply discredit such information due to a lack of trust in the information source but still aim to arrive at an accurate conclusion [1]. Though the processing goal (i.e., directional or accuracy) was not measured or manipulated in the current study, parsing out value and prior belief’s influence on ideological polarization may serve as an initial step to explicate the processing strategies people utilize when facing contentious issues. We recommend future research to utilize a similar scheme as the current study, but also consider how different processing goals would influence the results.

## 5. Conclusions

Climate change impacts are increasingly felt by people all over the world [1,2]. However, because people hold different political ideologies, environmental values, and beliefs in climate science, climate change communication practitioners need to tailor their message to achieve optimal persuasive effects. Highlighting the economic impacts of climate change in the U.S. may be especially useful when communicating to conservatives who value economic growth [11,12]. Differently, though public health threats imposed by climate change may seem alarming and universally appealing, they may also instigate more counterarguing among conservatives [4]. In addition to message framing, this study once again stresses the importance to monitor people’s belief in climate science because it is a persistent driver of ideological polarization in risk perception and policy support. From a broader perspective, this study attests to the importance of pinpointing sociopsychological factors that contribute to the increasingly polarized and politicized discussion on science issues such as climate change and the ongoing COVID-19 pandemic [2,6]. As people may respond differently to risk and science communication messages not only due to their ideological leaning, but other factors intrinsic to their values and beliefs, more strategic usage of message framing is necessary.

## Figures and Tables

**Table 1:** Regression models predicting risk perception and policy support with message frames, ideology, and their interaction (unstandardized regression coefficients).

Predictors	Risk Perception	Policy Support
Intercept	2.79	4.02
Issue ^1^	−0.03	0.7 *
Distance ^2^	0.14	0.32
Issue × Distance	−0.72 *	−1.31 **
Ideology	0.27 ***	0.35 ***
Ideology × Issue	0.01	−0.11
Ideology × Distance	−0.01	−0.03
Ideology × Issue × Distance	0.09	0.19 *
ANOVA	*F* (7, 845) = 47.42 ***	*F* (7, 845) = 33.8 ***
*R* ^2^	0.28	0.22
Adjusted *R*^2^	0.28	0.21

^1^ dummy-coded (0 = economy; 1 = public health); ^2^ dummy-coded (0 = close; 1 = far); * *p* < 0.05; ** *p* < 0.01; *** *p* < 0.001; significant coefficients are in bold.

**Table 2:** Regression models predicting risk perception and policy support with message frames, environmental value and their interaction (unstandardized regression coefficients).

Predictors	Risk Perception	Policy Support
Intercept	5.31	7.29
Issue ^1^	0.02	0.26
Distance ^2^	−0.19	−0.11
Issue × Distance	0.16	0.04
Environmental Value (EV)	−0.60 ***	−0.76 ***
EV × Issue	0.02	0.00
EV × Distance	0.13	0.13
EV × Issue × Distance	−0.22 *	−0.23
ANOVA	*F* (7, 846) = 70.78 ***	*F* (7, 846) = 76.97 ***
*R* ^2^	0.37	0.39
Adjusted *R*^2^	0.36	0.38

^1^ dummy-coded (0 = economy; 1 = public health); ^2^ dummy-coded (0 = close; 1 = far); * *p* < 0.05; *** *p* < 0.001; significant coefficients are in bold.

**Table 3:** Regression models predicting risk perception and policy support with message frames, belief in climate science and their interaction (unstandardized regression coefficients).

Predictors	Risk Perception	Policy Support
Intercept	1.38	2.44
Issue ^1^	0.24	0.76 *
Distance ^2^	0.47	0.24
Issue × Distance	−1.11 **	−1.35 *
Belief in Climate Science (BCS)	0.48 ***	0.58 ***
BCS × Issue	−0.02	−0.08
BCS × Distance	−0.06	−0.01
BCS × Issue × Distance	0.15 *	0.17
ANOVA	*F* (7, 846) = 114.59 ***	*F* (7, 846) = 92.31 ***
*R* ^2^	0.49	0.43
Adjusted *R*^2^	0.48	0.43

^1^ dummy-coded (0 = economy; 1 = public health); ^2^ dummy-coded (0 = close; 1 = far); * *p* < 0.05; ** *p* < 0.01; *** *p* < 0.001; significant coefficients are in bold.

**Table 4:** Direct and indirect effects of ideology on risk perception in four experimental conditions (unstandardized regression coefficients).

	Economy—Close		Economy—Far		Public Health—Close		Public Health—Far	
	Estimate ^1^	95% CI ^2^	Estimate ^1^	95% CI ^2^	Estimate ^1^	95% CI ^2^	Estimate ^1^	95% CI ^2^
Ideology → Risk Perception (direct effect)	0.03	(−0.04, 0.09)	**0.13**	**(0.06, 0.19)**	0.06	(−0.003, 0.13)	0.04	(−0.04, 0.12)
Ideology → Environmental value → Risk Perception	**0.06**	**(0.003, 0.12)**	0.04	(−0.01, 0.09)	**0.08**	**(0.02, 0.14)**	**0.09**	**(0.03, 0.15)**
Ideology → Science Belief → Risk Perception	**0.18**	**(0.12, 0.25)**	**0.13**	**(0.08, 0.18)**	**0.15**	**(0.08, 0.22)**	**0.19**	**(0.12, 0.26)**

^1^ Estimated direct and indirect effects with bootstrapped samples (N = 5,000); ^2^ 95% CI = bootstrapped 95% Confidence interval; significant coefficients are in bold.

**Table 5:** Direct and indirect effects of ideology on policy support in four experimental conditions (unstandardized regression coefficients).

	Economy—Close		Economy—Far		Public Health—Close		Public Health—Far	
	Estimate ^1^	95% CI ^2^	Estimate ^1^	95% CI ^2^	Estimate ^1^	95% CI ^2^	Estimate ^1^	95% CI ^2^
Ideology → Policy support (direct effect)	0.07	(−0.02, 0.16)	**0.12**	**(0.03**, **0.20**)	−0.05	(−0.13, 0.04)	−0.05	(−0.15, 0.06)
Ideology → Environmental value → Policy Support	**0.09**	**(0.02**, **0.17**)	0.06	(−0.01, 0.13)	**0.17**	**(0.11**, **0.23**)	**0.14**	**(0.08**, **0.22**)
Ideology → Science Belief → Policy Support	**0.19**	**(0.10**, **0.28**)	**0.20**	**(0.13**, **0.27**)	**0.13**	**(0.06**, **0.22**)	**0.24**	**(0.15**, **0.32**)

^1^ Estimated direct and indirect effects with bootstrapped samples (N = 5,000); ^2^ 95% CI = bootstrapped 95% Confidence interval; significant coefficients are in bold.

## Appendix A

Links to video stimuli.

- Close-distance, economy: https://youtu.be/zyYMdxUQag4
- Close-distance, public health: https://youtu.be/pn0Xrk5vr0U
- Far-distance, economy: https://youtu.be/NlTFpnlfIAc
- Far-distance, public health: https://youtu.be/4-swqGhqg0w
